# PET Radiopharmaceuticals

**Published:** 2011

**Authors:** Soraya Shahhosseini

PET (Positron Emission Tomography) is a powerful imaging technique through which quantitative information on the distribution of positron emitter labeled radiopharmaceuticals (PET radiopharmaceuticals) in the body can be realized. Positrons (β^+^) are positively charged beta particles. They are emitted when the atom is proton enriched. A positron has only a transient existence. After losing all of its kinetic energy, it interacts with an electron and is annihilated. Both the mass of positron and electron are converted to energy during annihilation and two 511 KeV photons are emitted at a 180° angle to each other. The PET is based on the coincidence detection of the two aforementioned photons. Coincidence detection is a powerful method enhancing sensitivity and dynamic-imaging capabilities of PET. PET camera systems contain a ring of detectors that encircles the patient. The data collected over many angles around the body axis of the patient is used to reconstruct the image of the activity distribution in slice or tomographic form. 

PET studies, like all nuclear medicine radioisotope emission procedures, yield images that represent the distribution or the pattern of uptaking radiopharmaceutical depending on the physiologic, pharmacologic, and biochemical state of the individual’s body. Thus, nuclear medicine procedures in general and PET procedures in particular are capable of providing information concerning how the body is functioning at a physiologic or biochemical level, whereas x-ray procedures such as computed tomography (CT) primarily depict human anatomy. At present, most of the new PET cameras are coupled to anatomical scanners. A PET/CT system allows the combination of functional and anatomic information and offers accurate matching of anatomic (CT) and functional (PET) images. Patients are imaged by both PET and CT in the same position of the patient, improving overall diagnostic utility of tomographic imaging methods. 

There are four positron emitting radioisotopes that are considered the biologic tracers, carbon-11, nitrogen-13, oxygen-15, and fluorine-18. ^11^C (t_1/2 _20.4 min), ^15^O (t_1/2 _2.1 min), and ^13^N (t_1/2 _10 min) are referred to as the essentials of life. They can be easily substituted directly into biomolecules without changing the properties of the molecule. ^18^F (t_1/2 _110 min) is not a normal constituent of biological molecules but can often be substituted for a hydroxyl group as in the case of deoxyglucose or can be substituted for a hydrogen atom in a molecule or placed in a position where its presence does not significantly alter the biological behavior of the molecule. Currently, there are four PET radiopharmaceuticals officially recognized by FDA: sodium fluoride (Na^18^F) for bone imaging, rubidium chloride (^82^RbCl) for assessment of regional myocardial perfusion in the diagnosis and localization of myocardial infarction, fluorodeoxyglucose (^18^FDG) for identifying the regions of abnormal glucose metabolism and primary and metastatic malignant diseases and ammonia (^13^NH_3_) for assessment of myocardial blood flow. ^18^FDG is currently the most widely used PET radiopharmaceutical in clinical oncology in addition to its clinical applications in cardiology and neurology. The application of PET in clinical oncology is increasing since many molecular targets relevant to cancer can be labeled with positron emitter radionuclides. 

The advantages of PET over traditional radionuclide imaging techniques include higher spatial resolution and sensitivity, quantification of activity, and synthesizing physiologically useful tracers. 

PET imaging requires expensive equipments including a cyclotron for radionuclide production, automated chemistry devices, purification instrumentation, and PET cameras. Though there are applications of PET imaging in cardiology and neurology, it seems that its greatest health application and benefit is in cancer diagnosis. 

At present, there are no PET centers in Iran. Research Institute for Nuclear Medicine, Tehran University of Medical Sciences and Masih Daneshvari Hospital, Shahid Beheshti University of Medical Sciences have purchased cyclotrons, PET cameras, and automated chemistry devices. Cyclotrons will be installed soon. In near future, these two centers will start producing ^18^FDG and other PET radiopharmaceuticals. We can expect PET imaging to have an increasing impact on health care and PET radiopharmaceuticals to be an active area of basic and clinical research and development. 

*Soraya Shahhosseini is currently working as an assistant professor of radiopharmacy at the Department of , School of Pharmacy, Shaheed Beheshti University of Medical Sciences, Tehran, Iran. She could be reached at the following e-mail address: Soraya.shahhosseini@gmail.com.*

